# MRI Evolution of a Patient with Viral Tick-Borne Encephalitis and Polymorphic Seizures

**DOI:** 10.3390/diagnostics12081888

**Published:** 2022-08-04

**Authors:** Carmen Adella Sirbu, Constantin Stefani, Marian Mitrică, Gabriela Simona Toma, Aurelian Emil Ranetti, Any Docu-Axelerad, Aida Mihaela Manole, Ion Stefan

**Affiliations:** 1 Department of Neurology, ‘Dr. Carol Davila’ Central Military Emergency University Hospital, 010242 Bucharest, Romania; 2 Department No. 5, University of Medicine and Pharmacy “Carol Davila”, 050474 Bucharest, Romania; 3 Clinical Neurosciences Department, University of Medicine and Pharmacy “Carol Davila”, 050474 Bucharest, Romania; 4 Department of Radiology, ‘Dr. Carol Davila’ Central Military Emergency University Hospital, 010242 Bucharest, Romania; 5 Department No. 2, University of Medicine and Pharmacy “Carol Davila”, 050474 Bucharest, Romania; 6 Department No. 4, Faculty of Medicine, ‘Ovidius’ University of Constanta, 900470 Constanta, Romania; 7 Department of Neurology, Clinical Ambulatory, ‘Dr. Carol Davila’ Central Military Emergency University Hospital, 010242 Bucharest, Romania; 8 Department of Infectious Diseases, ‘Dr. Carol Davila’ Central Military Emergency University Hospital, 010242 Bucharest, Romania; 9 Department of Medico-Surgical and Prophylactic Disciplines, Titu Maiorescu University, 031593 Bucharest, Romania

**Keywords:** tick-borne encephalitis (TBE), flavivirus, tick-borne encephalitis virus (TBEV), imaging, CSF, EEG, basal ganglia, MRI, polymorphic seizures

## Abstract

Some neurotropic viruses induce specific lesions in the deep structures, such as basal ganglia and thalamus. These anatomical structures play an important role in initiating and maintaining different types of epileptic seizures. We present the case of a 25-year-old male, transferred to our clinic one week after the onset of the symptomatology, with a recent history of traveling to Turkey and Egypt. At the moment of his hospital admission, his symptoms included altered consciousness, agitation, and seizures. Shortly after, his state worsened, requiring intubation. Viral tick-borne encephalitis diagnoses were favored by the CSF (cerebrospinal fluid) analysis, EEG (Electroencephalography), MRI (magnetic resonance imaging) images presenting symmetric hyper signal in the basal ganglia, and IgM antibodies for anti-tick-borne encephalitis. These lesions persisted for several weeks, and the patient’s seizures were polymorphic, originally generalized onset motor, generalized onset non-motor, and focal myoclonic. The patient achieved his independence, seizures decreasing both in intensity and frequency; the MRI images became almost normal. The reduction in antiepileptic doses was not followed by seizure recurrence.

The thalamus and basal ganglia significantly trigger and sustain various types of seizures, having a neuromodulatory role. Some viruses that invade the central nervous system induce specific lesions in the basal ganglia and thalamus. The Flaviviridae family is one of the most common sources of zoonoses. Its most important representatives are the Japanese encephalitis virus, the yellow fever virus, the West Nile virus, the hemorrhagic fever viruses, the Zika virus—all being transmitted by the Culex mosquito—and tick-borne encephalitis virus (TBEV), transmitted via tick bite. Mainly the basal ganglia and sometimes the thalamus are affected by cerebral involvement.

A 25-year-old patient with a known history of recent travel to Turkey and Egypt (countries where there have been recent cases of TBE) was admitted to a regional medical care unit for a significantly altered general status, headache, seizures, and fever and was transferred to our ward five days later.

At admission, the patient had a profoundly altered general condition. The brain CT was normal, and the MRI revealed slightly increased bilateral putaminal and caudate nucleus signal, with moderate bilateral putaminal restricted diffusion (Figure 1). Intercritical EEG recording with alpha background rhythm, average frequency 8 Hz and amplitude 28 µV, intricate with slow waves predominantly left frontotemporal with secondary bilateraleralization, was suggestive of a lesional substrate. In the clinical context, the patient’s seizures were polymorphic, originally generalized onset motor, generalized onset non-motor, and focal myoclonic. Unfortunately, video-EEG monitoring was not possible at that time. Slightly increased leukocytes were found in the blood tests, with 10.3% lymphocytes, 81.5% granulocytes, and 113 mg/dL glycemia. Dosing of anti-NMDA (N-methyl D-aspartate receptors), anti-GAD (glutamic acid decarboxylase), anti-GABA (gamma-aminobutyric acid), anti-neuronal, and anti-VGKC (voltage gated potassium channel-complex) antibodies were negative. A lumbar puncture was performed, obtaining a clear, colorless CSF with a glycorrhachia of 97 mg/dL, proteins of 51 mg/dL, 586 erythrocytes/mm^3^ (traumatic lumbar puncture), 27 leukocytes/mm^3^, lymphocytes 83%, and polymorphonuclears 17%. Common bacterial or viral pathogens were absent in the CSF using the PCR analysis panel for Staphylococcus, Streptococcus, Enterobacteriaceae, Enterococcus, Escherichia Coli, Haemophilus influenzae, Listeria monocytogenes, Neisseria meningitides, Streptococcus agalactiae, Streptococcus pneumonia, Cytomegalovirus, Enterovirus, Herpes simplex virus 1/2, Human herpesvirus 6, Human parechovirus, Cryptococcus neoformans/Gatti, and Varicella zoster virus. The immuno-enzymatic test IgM antibodies for the anti-tick-borne encephalitis virus was positive (over 0.8 ratio). Therefore, the diagnosis was tick-borne encephalitis. The treatment of the infection is mostly symptomatic, as there are currently no specific anti-TBEV agents. The patient required oro-tracheal intubation, which was maintained for 23 days. He continued to have generalized motor seizures and was sedated, presenting GCS 3, SOFA 4 (Sequential Organ Failure Assessment—mortality prediction in ED; SOFA 4 ≈ 7.0% mortality), APACHE 7 (Acute Physiology and Chronic Health Evaluation–mortality prediction in ED; APACHE 7 ≈ 10% mortality). Phenytoin and, later, valproic acid and levetiracetam were initiated for underlying seizures and psychotic disturbances.

At that time, after intubation, the second MRI revealed bilateral putaminal high signal intensity, on T2 and FLAIR sequences and low restricted diffusion in both putaminal regions, without enhancement in the bilateral caudate nucleus and putamen (Figure 2).

Six days later, MRI showed a persistent high T2 signal intensity in the posterior 2/3 of the putamen and normal signal intensity of the caudate nucleus, with symmetrical moderate restricted diffusion in the posterior 2/3 of the putamen and no restricted diffusion in the caudate nucleus (Figure 3).

After resuming unassisted breathing, the neurological examination showed an oriented patient with focal motor, generalized non-motor, and focal myoclonic seizures, being partially aware. Perceptual qualitative disorders such as simple and complex auditory hallucinations were highlighted; inappropriate behavior, voluntary and spontaneous hypoprosexia, accelerated rhythm and verbal fluency, no delusional ideation, and moderate depression due to the recognition of health problems were also noted. Olanzapine was introduced to treat his psychosis. Another lumbar puncture showed a glycorrhachia of 96 mg/dL and Crl proteins of 39 mg/dL.

MRI on day 45 revealed limited hyperintensity T2 in bilateral putamen in the posterior external region and minimal hyperintensity in the periphery of bilateral putamen on FLAIR. Minimal restricted diffusion persisted in the periphery of bilateral putamen (Figure 4).

On discharge, the patient presented normal signal intensity with only a discrete band of peripheral high signal intensity T2 wi/FLAIR in bilateral putamen but without restricted diffusion in the lenticular nucleus (Figure 5). The patient had a normal neurological examination and no seizure activity. Treatment with valproic acid 1200 mg, levetiracetam 2000 mg, and Olanzapine 5 mg was indicated at his discharge. One month later, he was fully recovered, without seizures, showing a normal EEG, and it was decided to gradually reduce the anticonvulsant and antipsychotic medication.

Tick-borne encephalitis is generated by TBEV infection, transmitted by ticks. There are more variants of Eurasiatic TBEV, such as the European variant, the Far Eastern variant, and the Siberian variant. Other variants of lesser importance have recently been discovered in the Balkans (Greece and Bulgaria), Turkey and Spain, and also in India and Egypt [1].

With an incubation period of 7 to 15 days, 30% of infections caused by TBEV remain asymptomatic. The disease’s evolution usually has two phases. Initially, most of the symptoms are flu-like, such as headache or myalgia, and they can last for 4–7 days or even up to 15 days [2]. In a minority of cases, after a short afebrile period, the encephalitic phase begins, and the symptoms worsen, affecting the nervous system: myelitis, meningitis, or encephalitis, depending on the location of the lesion [3,4].

The role of the basal ganglia in initiating and maintaining seizures is controversial. While some authors consider the frontal and temporal lobes to be epileptogenic sources, others incriminate the basal ganglia. The role of the latter is well established: it is known that the efferents of the direct and indirect pathways exert an inhibitory activity on the anterior and lateral ventral thalamus, filtering the exaggerated motor activity from the cortex. Multiple studies demonstrated the above [5,6,7,8,9]. Regarding generalized nonmotor seizures, the basal ganglia have a better-established role. The main pathways involved in controlling seizures are excitatory pyramidal neurons, subthalamic nucleus, spinal medial neurons, and specific relay nuclei [10]. Various studies suggest that stimulation of the striatum, external globus pallidus, subthalamic nucleus, and reticulated substantia nigra or thalamus by deep brain stimulation may decrease epileptiform activity in generalized nonmotor seizures. Recent MRI studies suggested that substantia nigra atrophy, along with metabolic and blood flow abnormalities, are responsible for decreased antiepileptic inhibitory activity [11,12]. Pars compacta degeneration plays a role in the onset of epilepsy [8]. There was a link between damage to the basal ganglia and initiation—then continuation—of polymorphic seizures.

Most people infected with TBEV recently traveled to areas known to be at high risk. Turkey, Egypt, and even Romania are countries where there were recently reported cases of infections caused by TBEV.

Our patient did not recall being possibly bitten by a tick. A minority of cases obtain the virus from low-risk areas, probably through non-vector transmission. In the case of our patient, the onset of encephalitis was clinically dramatic, with acute deterioration of consciousness and seizures. It is important to emphasize the characteristic prodrome consisting of headache, altered general condition, and chills. We would like to mention that the patient had never been vaccinated against the yellow fever virus. The immunoassay test could be falsely positive due to cross-linked reactions with other flaviviruses. The patient had not been vaccinated against TBE either. We would like to emphasize the importance of vaccination in endemic areas using the FSME Immun or other anti-TBEV vaccines.

MRI imaging revealed symmetrical bilateral damage to the basal ganglia and the temporally overlapped remission of the lesions with the improvement of symptoms. Other pathologies that can affect the basal ganglia symmetrically and have a specific MRI appearance were excluded (Table 1).

We concluded that MRI is an important non-invasive tool for the follow-up and prognosis of viral meningitis.

## Figures and Tables

**Figure 1:**
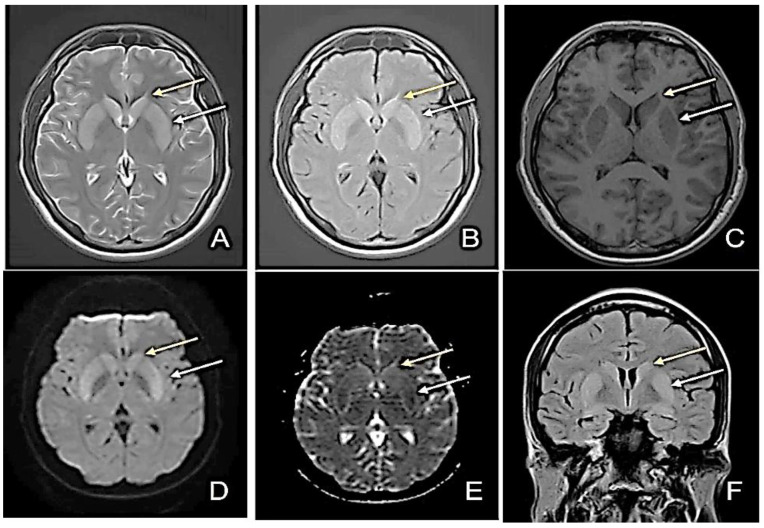
First Magnetic Resonance Imaging (MRI)—day 1 at the admission to our clinic (D1). (**A**) Axial T2-weighted image sequence: slightly increased bilateral putaminal and caudate nucleus signal; (**B**) Axial FLAIR sequence: increased bilateral putaminal and caudate nucleus signal; (**C**) Axial T1-weighted image sequence: slightly decreased bilateral putaminal and caudate nucleus signal; (**D**) Diffusion-weighted image sequence (DWI); (**E**) Apparent diffusion coefficient map (ADC): moderate bilateral putaminal restricted diffusion; (**F**) Coronal FLAIR image: slightly increased bilateral putaminal (white arrow) and caudate nucleus signal (yellow arrow).

**Figure 2:**
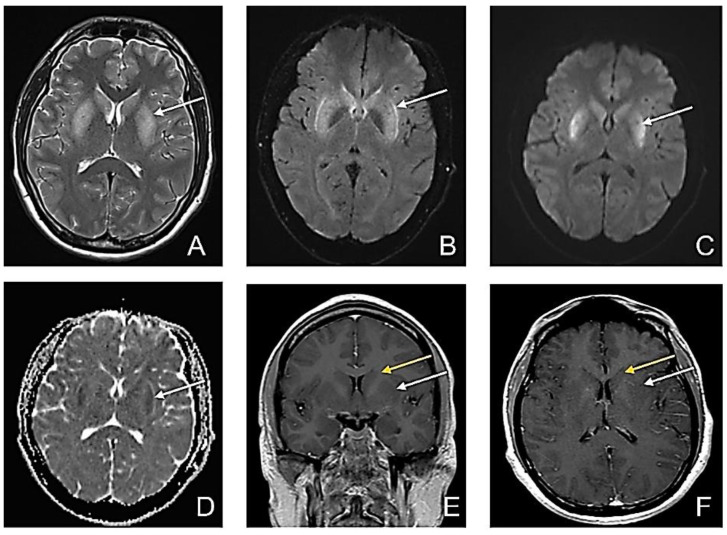
Second MRI-D5. (**A**) Axial T2-weighted image sequence: bilateral putaminal high signal intensity; (**B**) Axial FLAIR sequence: bilateral putaminal high signal intensity; (**C**) Axial DWI: high signal intensity in putaminal regions; (**D**) Axial ADC map: low ADC in the putaminal regions suggestive of restricted diffusion in both putaminal regions (white arrow); (**E**) Contrast-enhanced Coronal T1wi; and (**F**) Contrast-enhanced Axial T1wi: no enhancement in the bilateral caudate nucleus (yellow arrow) and putamen (white arrow) with slightly decreased signal in bilateral putamen.

**Figure 3:**
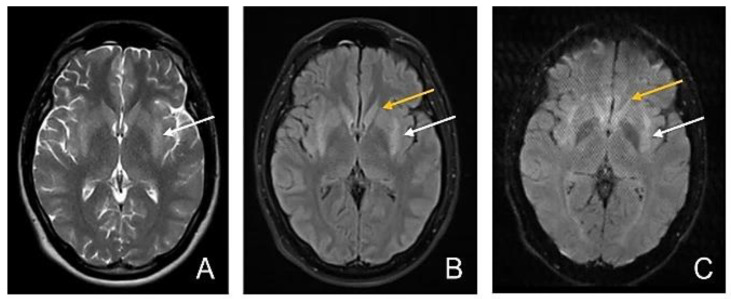
Third MRI—D11. (**A**) Axial T2-wi sequence: persistent high T2 signal intensity in posterior 2/3 of the putamen; (**B**) Axial FLAIR image: Symmetrical hyperintensity of the posterior 2/3 of the putamen (white arrow) and normal signal intensity of the caudate nucleus (yellow arrow); (**C**) Axial DWI: persistent symmetrical moderate restricted diffusion in the posterior 2/3 of the putamen (white arrow) and no restricted diffusion in the caudate nucleus (yellow arrow).

**Figure 4:**
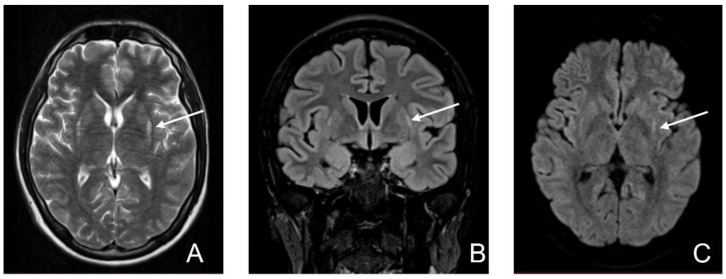
Fourth MRI (D45). (**A**) T2-wi sequence: limited hyper signal in bilateral putamen in the posterior external region; (**B**) Cor FLAIR image: minimal hyperintensity in the periphery of bilateral putamen; (**C**) Axial DWI: minimal restricted diffusion persistent in the periphery of bilateral putamen (white arrow).

**Figure 5:**
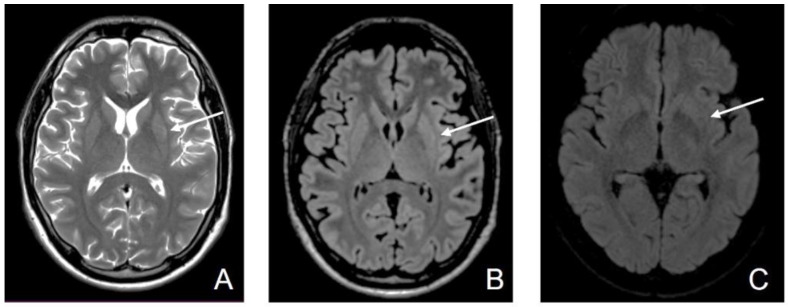
Fiveth MRI (D63). (**A**) Axial T2-wi; (**B**) Axial FLAIR: normal signal intensity with only a discrete band of peripheral high signal intensity T2 wi/FLAIR in bilateral putamen; (**C**) Axial DWI: there is no more restricted diffusion in the lenticular nucleus (white arrow).

**Table 1:** MRI abnormalities of the basal ganglia found in different pathologies.

Disease	T1w Sequence	T2w Sequence	FLAIR	DWI
Hypoxic-ischemic encephalopathy [13,14]	In severe cases, hyperintensities may be encountered due to the accumulation of denatured proteins, secondary to necrosis	In the first two weeks, hyperintensities and swelling of the affected areas due to inflammation of the affected grey matter can be observed	Hyperintensities in the affected areas	Increased DWI signal and low ADC, suggestive of restricted diffusion
Leigh disease [15,16]	Decreased T1wi signal in the areas with T2 hyperintensities; rarely, T1 hyperintensities may be encountered	Hyperintensities in the following structures: basal ganglia (especially putamen), brain stem, periaqueductal brain matter, medulla, midbrain, thalami	Hyperintensities such as T2wi	Restricted diffusion may be seen in the acute setting
Hypoglycemic encephalopathy [17,18,19]	Hyposignal (usually bilateral) in the cerebral cortex, internal capsule, hippocampus, or basal ganglia	Hyperintensity in one or more of the T1w-mentioned structures	Hyperintensities such as T2wi	It is a sensitive sequence showing reversible diffusion restriction from the early hours
Wilson’s disease [20,21]	Hypointensities in deep grey matter structures	Hyperintensities in deep grey matter structures, especially in the putamen and bilateral thalami. Giant panda sign (increased signal intensity in the midbrain tegmentum with the normally hypointense red nucleus	Giant panda sign.	Restricted diffusion may be the first imaging change
Toxic substances [22,23]	It depends on the substance involved	Generally, T2 hyperintensities in the affected areas	Generally, FLAIR hyperintensities in the affected areas Confluent, symmetrical lesions that may involve the corpus callosum	Confluent and symmetrical restricted-diffusion lesions that may involve the corpus callosum or white matter
Hepatic encephalopathy [24,25]	Hyperintensities in the basal ganglia, subthalamic regions, and globus pallidus	High signal intensities involving the hemispheric corticospinal tract and focal hyperintense T2 lesions in subcortical hemispheric white matter	Hyperintensities such as T2wi	Increase mean diffusivity in the affected areas
Non-ketonic hyperglycemia [26,27]	Hyperintensities in the basal ganglia (more often, the putamen or caudate nucleus are involved)	Hyperintensities in the regions described in the T1w sequence	Sometimes subcortical hypointensity and cortical hyper signal	Basal ganglia hyperintensity

## Data Availability

Not applicable.
